# Case Report: Definite DRESS following phenobarbital exposure with secondary HLH and DAT-positive hemolysis during high-dose IVIG treatment in a child

**DOI:** 10.3389/fimmu.2026.1907799

**Published:** 2026-09-08

**Authors:** Lingmei Zhang, Siyu Lei, Daiyu Zhang, Feng Chen, Qian Wan, Hong Wang, Zhongjin Xu, Chongjun Wu

**Affiliations:** 1 Department of Hematology, Jiangxi Provincial Children’s Hospital, Nanchang, China; 2 Jiangxi Medical College, Nanchang University, Nanchang, China

**Keywords:** case report, DAT-positive hemolysis, dress, intravenous immunoglobulin, secondary hemophagocytic lymphohistiocytosis

## Abstract

**Background:**

Drug reaction with eosinophilia and systemic symptoms (DRESS) is a severe delayed drug hypersensitivity reaction that may overlap with secondary hemophagocytic lymphohistiocytosis (HLH). Direct antiglobulin test (DAT)-positive hemolysis during intravenous immunoglobulin (IVIG) treatment creates an attribution problem because disease-associated autoimmune hemolysis and treatment-related passive red-cell sensitization or hemolysis may present similarly.

**Case presentation:**

A 13-year-and-5-month-old boy developed fever and a generalized pruritic eruption following phenobarbital exposure. A point-by-point RegiSCAR assessment yielded 6 points, classifying the presentation as definite DRESS. He fulfilled five HLH-2004 criteria, consistent with secondary HLH. The patient was blood group AB and RhD-positive, received no red-cell transfusions, and received 87.5 g of IVIG (approximately 2.0 g/kg). After 77.5 g had already been administered, dark urine was observed and DAT sampling was performed on hospital day (HD) 5 before the final 10 g IVIG infusion. Hemoglobin fell from 119 g/L on HD 2 to 86 g/L on HD 5 and reached 83 g/L on HD 7; indirect bilirubin increased, and DAT was immunoglobulin G (IgG) positive (1+) and C3d negative. DAT weakened on HD 9 and became negative on HD 13. Neither pre-IVIG DAT nor red-cell antibody screening was performed, and no subsequent antibody elution or specificity testing was available. Hemolysis profiling was further limited by the absence of haptoglobin measurements, peripheral-smear assessment, cold-antibody testing, and serial reticulocyte measurements. Withdrawal of suspected drugs, high-dose methylprednisolone followed by tapering, and supportive care were followed by sustained recovery without red-cell transfusion or HLH-specific cytotoxic therapy.

**Conclusion:**

This case suggests that, in a child with definite DRESS following phenobarbital exposure and secondary HLH, DAT-positive hemolysis is temporally associated with high-dose IVIG exposure, although the underlying mechanism remains unclear. Therefore, in similar clinical scenarios, we suggest documenting blood group, pre-IVIG DAT and antibody screening, and cumulative dose prospectively.

## 1 Introduction

Drug reaction with eosinophilia and systemic symptoms (DRESS) is a delayed severe cutaneous adverse reaction characterized by fever, widespread eruption, hematologic abnormalities, lymphadenopathy, and variable internal-organ injury (1, 2). Because no single feature is diagnostic, the RegiSCAR framework integrates clinical and laboratory findings and classifies cases as no, possible, probable, or definite DRESS (1). Although widely used in pediatric practice, RegiSCAR has not been formally validated in children (3). Aromatic antiseizure medications, including phenobarbital, are recognized as suspected triggers. In children, early DRESS may resemble infection, Kawasaki disease, Stevens–Johnson syndrome/toxic epidermal necrolysis (SJS/TEN), or other systemic inflammatory disorders.

Secondary hemophagocytic lymphohistiocytosis (HLH) is a hyperinflammatory syndrome associated with uncontrolled immune activation and progressive organ dysfunction. The HLH-2004 framework permits diagnosis when five of eight criteria are fulfilled (4). Because HLH-2004 features are not disease-specific, their fulfillment should be interpreted in clinical context and alongside evaluation of underlying contributors (5). Severe DRESS and secondary HLH may share persistent fever, hepatosplenomegaly, cytopenias, liver injury, hyperferritinemia, and coagulation abnormalities, and their co-occurrence has been reported in adults and children (6–8). Criteria-based assessment is therefore essential, and marrow hemophagocytosis should not substitute for evaluation of the complete diagnostic profile.

Direct antiglobulin test (DAT)-positive hemolysis introduces a separate attribution problem. DAT positivity demonstrates immunoglobulin and/or complement coating of red cells but does not identify the antibody source or independently establish autoimmune hemolytic anemia (AIHA) (9, 10). High-dose intravenous immunoglobulin (IVIG) can passively transfer anti-A or anti-B antibodies and may cause DAT positivity or clinically significant hemolysis, particularly in non-O recipients receiving high cumulative doses (11–15). We report a child with definite DRESS following phenobarbital exposure who fulfilled five HLH-2004 criteria and developed DAT-positive hemolysis during high-dose IVIG treatment. This report focuses on the evolving immune phenotype of severe pediatric DRESS, including secondary HLH and DAT-positive hemolysis, while addressing the diagnostic challenge of interpreting hemolysis in the context of IVIG exposure and incomplete baseline immunohematologic evaluation.

## 2 Case presentation

A 13-year-and-5-month-old boy weighing 44 kg was transferred on 11 March 2026, which was designated hospital day (HD) 1, because of 8 days of recurrent fever and 5 days of generalized rash. He had been somnolent for 2 days before transfer, with somnolence lasting approximately 5 days in total. He had epilepsy and had received valproate 500 mg twice daily for approximately 6 months before admission. Phenobarbital 90 mg twice daily had been started approximately 10 weeks before admission. Fever began 8 days before admission, after approximately 63 days of phenobarbital exposure, and phenobarbital was stopped that day. A pruritic erythematous maculopapular eruption began on the abdomen 5 days before admission and spread from the trunk to the limbs. Phenobarbital was not reintroduced, and no lymphocyte transformation test, patch test, or other formal drug-causality testing was performed.

Before transfer, short courses of amoxicillin and ceftazidime were initiated after fever onset but before or around rash onset. During the 2 days before admission, Kawasaki disease and infection were suspected, and the patient received methylprednisolone 40 mg every 12 h, piperacillin-tazobactam 4.5 g every 8 h, aspirin, and heparin. A total of 32.5 g of 5% human immunoglobulin was infused on the day before admission. Persistent high fever and progressive systemic manifestations prompted transfer. Valproate was discontinued on HD 1, and antiseizure therapy was changed to levetiracetam 440 mg twice daily. Cefoperazone-sulbactam was administered from HD 1 to HD 5. The complete drug, treatment, and event chronology is provided in **Supplementary Table 1**.

The reported peak temperature was 41 °C. The eruption involved the face, trunk, back, and extremities and exceeded 50% of the body surface area. It was partly confluent and infiltrated, with facial edema, focal purpuric change, subsequent desquamation, and residual hyperpigmentation; cutaneous resolution required approximately 30 days. Cervical nodes were enlarged bilaterally, measuring up to 27 × 9 mm, and an inguinal node measured approximately 12 × 10 mm. Admission findings included cracked lips with focal bleeding, angular erosions, mild strawberry tongue, pharyngeal congestion, and bilateral nonexudative conjunctival congestion. Mild intraoral erosions with an adherent white coating were documented on HD 4. There was no blistering, epidermal detachment, extensive mucosal sloughing, corneal involvement, genital involvement, or positive Nikolsky sign.

At admission, hemoglobin was 110 g/L, platelets were 120 × 10^9^/L, white-cell count was 8.45 × 10^9^/L, and absolute eosinophil count was 0.73 × 10^9^/L. The absolute eosinophil count peaked at 1.50 × 10^9^/L on HD 7. Alanine aminotransferase and aspartate aminotransferase peaked at 123 and 290 U/L, respectively, 2 days before admission. Ferritin was >1,650 ng/mL on the day before admission and peaked at 2,595 ng/mL on HD 3. On HD 1, triglycerides were 3.21 mmol/L, fibrinogen was 1.08 g/L, and soluble interleukin-2 receptor was 6,667 U/mL (reference range, 228–724 U/mL). Computed tomography on HD 2 confirmed splenomegaly, multiple mildly enlarged abdominal nodes, a small pneumatocele-like change in the right lower lobe, and small bilateral pleural effusions without radiologic pneumonia or interstitial lung disease. Bone marrow aspiration showed approximately 20% eosinophils and occasional histiocytic phagocytosis, without evidence of leukemia or lymphoma. Complete longitudinal laboratory data are provided in **Supplementary Table 2**, and imaging, marrow, and genetic findings are summarized in **Supplementary Table 3**.

The cumulative IVIG dose was 87.5 g (approximately 2.0 g/kg): 32.5 g on the day before admission, 15 g on each of HDs 2–4, and 10 g on HD 5. All preparations were documented as 5% human immunoglobulin. Commercial product names and lot numbers could not be retrieved. The patient was blood group AB and RhD-positive and received no red-cell transfusions.

**Figure 1:**
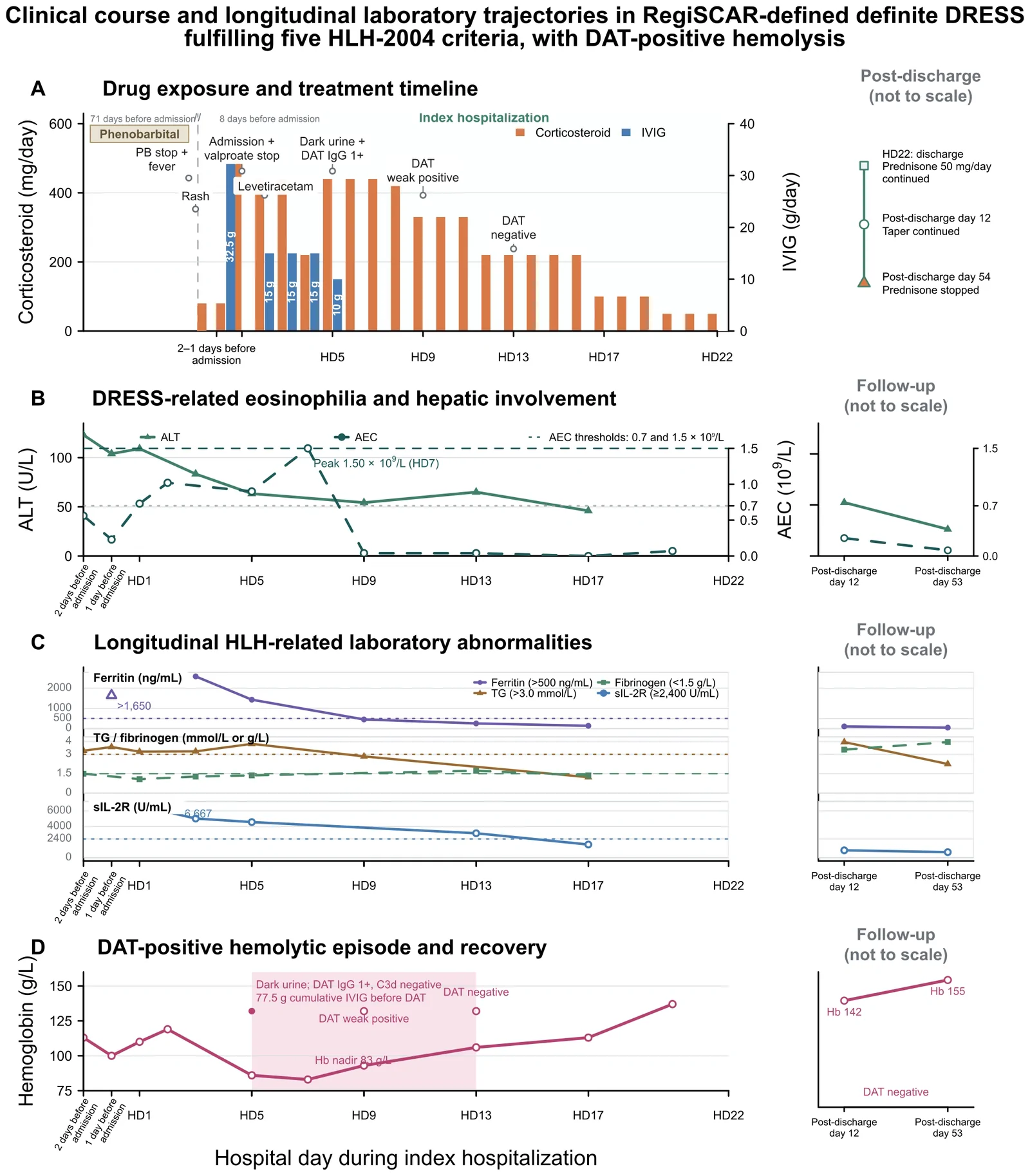
Clinical course and longitudinal laboratory trajectories in a patient with definite DRESS fulfilling five HLH-2004 criteria, with DAT-positive hemolysis. **(A)** Clinical timeline showing phenobarbital exposure and withdrawal, fever and rash onsets, admission, valproate discontinuation, corticosteroid treatment, IVIG administration, dark urine, DAT sampling and evolution, discharge, and follow-up. Dark urine was observed and DAT sampling was performed before the 17:15 IVIG infusion on HD 5, after a cumulative IVIG dose of 77.5 g. **(B)** Absolute eosinophil count and hepatic involvement. **(C)** HLH-related markers during the hyperinflammatory window, including ferritin, triglycerides, fibrinogen, and sIL-2R. **(D)** Hemoglobin trajectory, DAT evolution, and recovery.

On HD 5, dark urine was observed and DAT sampling was performed before the final 10 g IVIG infusion, but 77.5 g had already been administered. The DAT was immunoglobulin G (IgG)-positive (1+) and C3d-negative, and the indirect antiglobulin test was negative. Urinalysis that day showed dark urine, trace blood, and 10 red cells/µL. Hemoglobin fell from 119 g/L on HD 2 to 86 g/L on HD 5 and reached 83 g/L on HD 7. On HD 5, total bilirubin was 23.4 µmol/L, indirect bilirubin was 14.4 µmol/L, and lactate dehydrogenase was 1,385 U/L; lactate dehydrogenase had already been 1,767.62 U/L at admission. The only reticulocyte result, obtained on HD 1, was 0.39% (15.64 × 10^9^/L). DAT remained weakly IgG positive on HD 9 and became negative on HD 13, when urine was clear and hemoglobin had risen to 106 g/L. No pre-IVIG DAT or red-cell antibody screen, antibody elution or specificity testing, haptoglobin measurement, peripheral-smear assessment, cold-antibody testing, serial reticulocyte measurements during the hemolytic window, or urine free hemoglobin/myoglobin testing was available (**Figure 1**).

**Figure 2:**
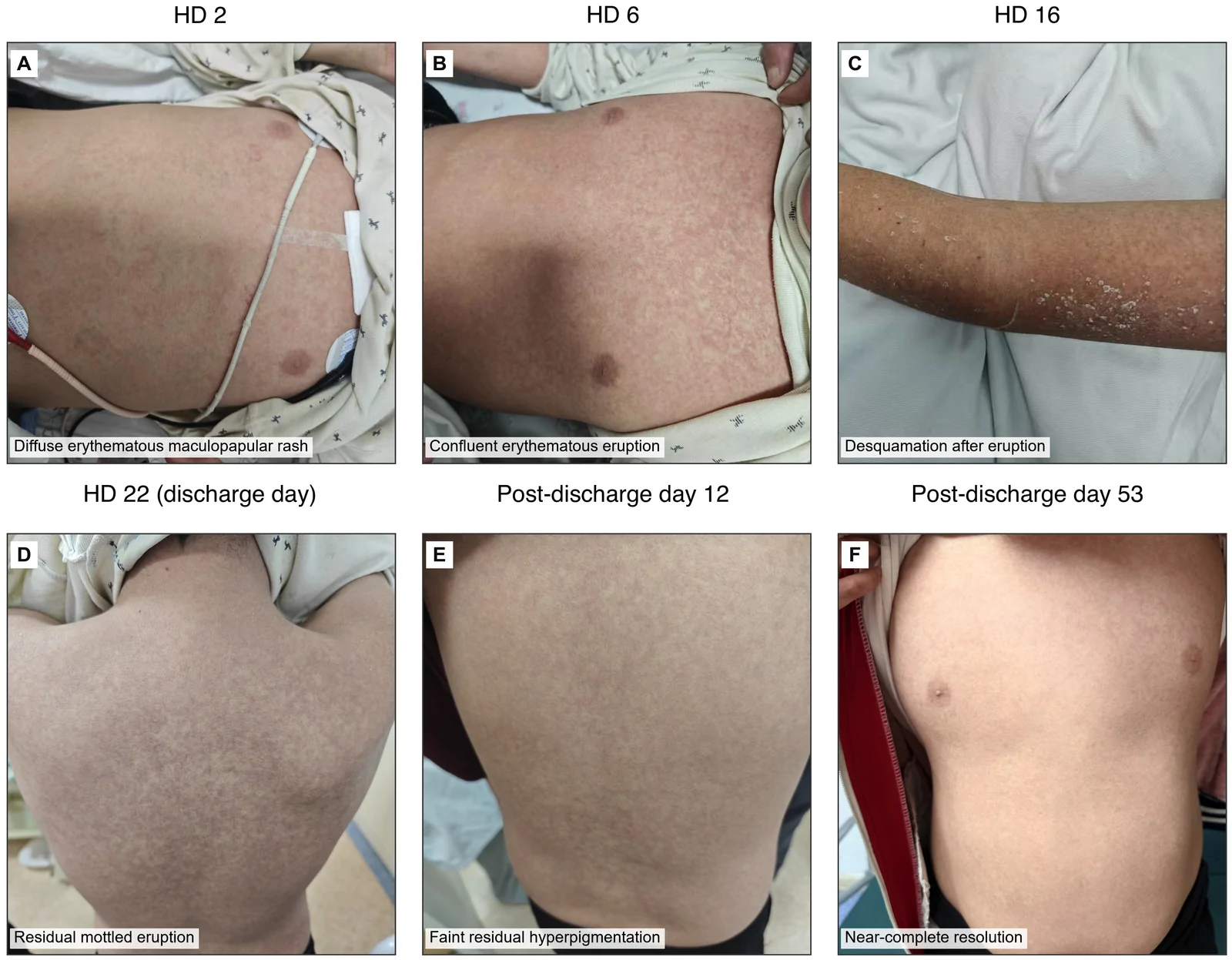
Serial cutaneous evolution. Representative de-identified clinical photographs show the eruption and recovery: **(A)** HD 2; **(B)** HD 6; **(C)** HD 16; **(D)** HD 22 (discharge day); **(E)** post-discharge day 12; and **(F)** post-discharge day 53. Only cropping and proportional resizing were applied. Written consent from the patient’s guardian was obtained for publication of clinical images.

Methylprednisolone was administered at 500 mg on HD 1, 440 mg/day on HD 2–3, and 220 mg on HD 4. Recurrence of fever and rash after this reduction prompted re-escalation to 440 mg/day on HD 5–8, followed by gradual tapering. Hydration and urine alkalinization were provided during the dark-urine episode. Oral prednisone 50 mg/day began on HD 20. The patient was discharged on HD 22 with a plan to reduce prednisone by 5 mg every 5 days; levetiracetam was continued. Etoposide, cyclosporine, anakinra, and ruxolitinib were not used. On post-discharge day 12, he had no fever, rash, or dark urine; hemoglobin was 142 g/L, ferritin was 102.8 ng/mL, and DAT was negative. On post-discharge day 53, he remained clinically well, with hemoglobin 155 g/L, ferritin 42.3 ng/mL, normalized liver tests, and negative DAT. Glucocorticoids were stopped on post-discharge day 54. Whole-exome sequencing did not identify pathogenic or likely pathogenic variants explaining the clinical phenotype. Serial cutaneous evolution is shown in **Figure 2**.

## 3 Diagnostic assessment and differential diagnosis

**Figure 3:**
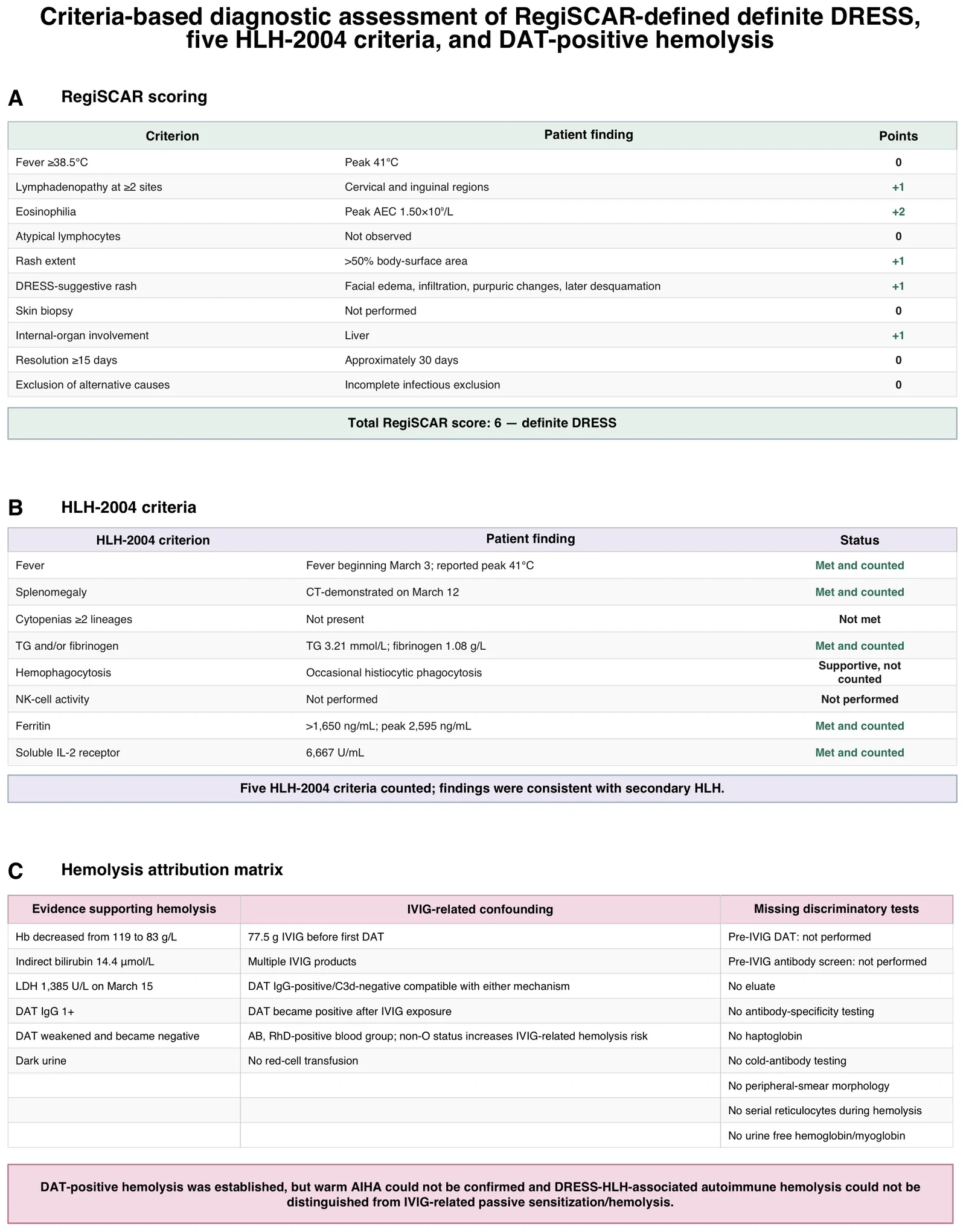
Criteria-based diagnostic assessment of definite DRESS, five HLH-2004 criteria, and DAT-positive hemolysis. **(A)** Point-by-point RegiSCAR assessment showing a total score of 6 and classification as definite DRESS. Phenobarbital was the most temporally plausible suspected trigger, but formal drug-causality testing was not performed. **(B)** Secondary HLH was supported by meeting five HLH-2004 criteria. Cytopenias did not meet the two-lineage threshold, natural killer cell activity was not assessed, and occasional marrow hemophagocytosis was treated as supportive morphology rather than counted as a sixth criterion. **(C)** Evidence supporting DAT-positive hemolysis and limitations precluding mechanistic attribution. Pre-IVIG direct antiglobulin testing and antibody screening, antibody elution and specificity testing, haptoglobin measurement, peripheral blood-smear morphology, cold-antibody testing, and serial reticulocyte counts during the hemolytic period were unavailable. Accordingly, the findings support DAT-positive hemolysis, with the underlying mechanism remaining indeterminate.

The RegiSCAR score was 6: lymphadenopathy in at least two anatomic regions (+1), a peak absolute eosinophil count of 1.50 × 10^9^/L (+2), rash involving >50% body surface area (+1), a DRESS-suggestive rash with facial edema and subsequent desquamation (+1), and hepatic involvement (+1). Fever and resolution beyond 15 days added no points; atypical lymphocytes were absent, skin biopsy was not performed, and incomplete exclusion of alternative causes was scored 0. The classification was definite DRESS (**Figure 3A**; **Supplementary Table 4**). Phenobarbital was the most temporally plausible suspected trigger. Valproate exposure was substantially longer, whereas short-course antibiotics were initiated only after fever onset and partly before or around rash onset; their short latencies made them less plausible as the primary DRESS trigger, although a modifying or contributory role could not be completely excluded.

Five HLH-2004 criteria were fulfilled: fever, computed tomography-confirmed splenomegaly, hypertriglyceridemia and/or hypofibrinogenemia, hyperferritinemia, and elevated soluble interleukin-2 receptor. Cytopenias did not meet the two-lineage threshold, natural killer cell function was not assessed, and occasional marrow phagocytosis was treated as supportive morphology rather than counted as a sixth criterion. The profile was consistent with secondary HLH in the setting of DRESS (**Figure 3B**; **Supplementary Table 5**).

The hemoglobin decline, indirect hyperbilirubinemia, dark urine, and IgG-positive DAT supported DAT-positive hemolysis. IVIG-related passive sensitization or hemolysis remained plausible because the patient was blood group AB and had received 77.5 g before the first DAT; disease-associated autoimmune hemolysis was also possible during active DRESS-HLH. The absence of baseline immunohematology and antibody characterization, together with incomplete hemolysis profiling, prevented distinction between these mechanisms. Hemoglobinuria was not laboratory-confirmed. The final classification was DAT-positive hemolysis temporally associated with both DRESS-HLH and preceding high-dose IVIG exposure (**Figure 3C**; **Supplementary Table 6**).

Available findings did not support active Epstein–Barr virus infection: blood and throat-swab polymerase chain reaction tests were negative on HD 1, and repeat blood polymerase chain reaction tests were negative on post-discharge days 12 and 53. Post-IVIG serology showed negative immunoglobulin M (IgM) to viral capsid antigen (VCA), positive VCA-IgG, and negative early antigen (EA)-IgG and Epstein–Barr nuclear antigen (EBNA)-IgG and was interpreted cautiously because of possible passive-antibody interference (16). Cytomegalovirus polymerase chain reaction was negative, but serology was not performed; human herpesvirus 6 reactivation was not assessed. A single post-IVIG *Mycoplasma pneumoniae* total-antibody titer of 1:80, below the local acute-infection threshold of 1:160 and in the absence of molecular testing, isotype-specific serology, paired titers, cold-agglutinin testing, or pneumonia, was insufficient to establish acute infection. Qualitative post-IVIG *Chlamydia pneumoniae* immunoglobulin M and G positivity, without polymerase chain reaction or paired serology, was also insufficient. An initial blood culture grew *Staphylococcus epidermidis*, but a repeat culture was negative, and the clinical course did not support persistent bacteremia; earlier antibiotic exposure nevertheless limited culture sensitivity. These infections were not supported by the available evidence but could not be completely excluded (**Supplementary Table 7**).

Kawasaki disease became less likely after reassessment because of the patient’s age, normal coronary imaging, erythrocyte sedimentation rate of 9 mm/h, eosinophilia, drug chronology, and persistent fever after initial IVIG. Systemic juvenile idiopathic arthritis/macrophage activation syndrome was not supported because arthritis, arthralgia, joint swelling, and a typical fever-synchronous evanescent salmon rash were absent; rheumatology evaluation did not support the diagnosis. SJS/TEN was disfavored by the absence of blistering, epidermal detachment, extensive mucosal sloughing, and a positive Nikolsky sign. Sepsis was considered initially but not retained as the final diagnosis because there was no shock, persistent bacteremia, or convincing microbiologic focus, and antibiotics produced no clear response. Bone marrow showed no evidence of leukemia or lymphoma.

## 4 Discussion

Definite DRESS was supported by a RegiSCAR score of 6, and five HLH-2004 criteria were fulfilled. The subsequent hemolysis was clinically meaningful, but its immune mechanism remained indeterminate. Previous reports have described the coexistence of DRESS and HLH (6–8, 17). In this case, the additional diagnostic difficulty was that DAT-positive hemolysis occurred after substantial exposure to high-dose IVIG.

The approximately 63-day phenobarbital latency and the eruption shortly after withdrawal made phenobarbital the most temporally plausible suspected trigger (1, 2). However, RegiSCAR classifies the syndrome rather than proving the culprit drug. Longer valproate exposure, shorter antibiotic latencies, and the absence of rechallenge or drug-specific testing precluded definitive attribution; accordingly, we use “following phenobarbital exposure” rather than a definitive drug-induced formulation.

DRESS and HLH share fever, liver dysfunction, hematologic abnormalities, hyperferritinemia, and coagulation disturbances (6–8). Here, the combined computed tomography-confirmed splenomegaly, triglyceride/fibrinogen abnormalities, ferritin elevation, and markedly increased soluble interleukin-2 receptor met the formal HLH-2004 threshold; occasional marrow phagocytosis was supportive but was not counted. Recovery without HLH-specific cytotoxic therapy does not negate the five fulfilled criteria or establish which component of the overlapping inflammatory phenotype predominated.

The major uncertainty concerned the DAT-positive hemolysis. IVIG-associated hemolysis generally emerges within days of exposure, with a commonly used risk window of up to 10 days; the risk is concentrated among non-O recipients, particularly those with blood groups A and AB (12–15). By contrast, warm autoimmune hemolytic anemia requires evidence that an autoantibody is driving hemolysis and may persist or relapse independently of further exogenous antibody exposure (9, 10). In this child, blood group AB, approximately 2.0 g/kg cumulative IVIG, onset after 77.5 g, and subsequent loss of DAT reactivity were compatible with an IVIG-related process, but no single finding was discriminatory because active DRESS-HLH and corticosteroid treatment overlapped.

The IgG-positive, C3d-negative DAT pattern and subsequent loss of reactivity could occur with either passive antibody exposure or an autoantibody. No red-cell transfusion had occurred, but no pre-IVIG DAT or antibody screen established the baseline, and no elution or antibody-specificity testing distinguished an ABO isoagglutinin from a broader autoantibody. Hemoglobin decline and indirect bilirubin elevation supported hemolysis; however, lactate dehydrogenase was already elevated, the only reticulocyte measurement preceded the event, and haptoglobin, smear morphology, and urine free hemoglobin were unavailable. Thus, DAT-positive hemolysis is supported, whereas warm AIHA is not established (9, 10).

Repeated negative Epstein–Barr virus polymerase chain reaction results and a negative cytomegalovirus polymerase chain reaction result did not support active infection, but post-IVIG serology and the absence of human herpesvirus 6 testing left residual uncertainty. Single post-IVIG *Mycoplasma* and *Chlamydia pneumoniae* serology results could not establish acute infection. These gaps justify conservative RegiSCAR scoring for alternative-cause exclusion and prevent attribution of the HLH-range inflammation or hemolysis to a viral mechanism (2, 17). Chronology, imaging, microbiology, cardiac assessment, and marrow findings made the remaining inflammatory and malignant alternatives less likely.

Recovery followed withdrawal of suspected drugs, corticosteroids, completion of IVIG, and supportive care. Because these interventions overlapped, treatment response cannot identify the mechanism of hemolysis or the independent effect of IVIG on DRESS-HLH. A strength of this report is the hospital-day–based reconstruction of drug exposure, IVIG dosing, serial DAT results, and longitudinal recovery. Additional limitations include the single-case design, the absence of a skin biopsy or validated drug-specific causality assessment, incomplete virologic and immunohematologic evaluation, the absence of functional natural killer cell testing, and unavailable IVIG lot data.

In similar patients, we suggest documenting blood group, pre-IVIG DAT and antibody screening, IVIG product and lot details, and cumulative dose prospectively (9–15). Serial hemoglobin, reticulocyte, bilirubin, lactate dehydrogenase, haptoglobin, urinalysis, and smear findings should be recorded over the same period (9–15). If DAT-positive hemolysis develops, timely antibody elution and specificity testing may help distinguish disease-associated autoimmunity from passively transferred antibodies (9, 10). In children, structured long-term follow-up is also warranted because delayed autoimmune sequelae, particularly thyroid disease and type 1 diabetes mellitus, have been reported after DRESS (3, 18).

## 5 Conclusion

This case illustrates how severe pediatric DRESS may evolve with overlapping inflammatory and hematologic manifestations, including secondary HLH and DAT-positive hemolysis. The coexistence of these features suggests that severe DRESS may represent a state of complex immune dysregulation in which distinct hematologic abnormalities emerge over the course of the disease. However, because hemolysis developed after substantial high-dose IVIG exposure and baseline immunohematologic testing and antibody characterization were unavailable, DAT positivity should be interpreted in the context of both the inflammatory phenotype and treatment timeline rather than as evidence of autoimmune hemolysis alone. Baseline immunohematologic evaluation and serial monitoring of inflammatory and hemolysis markers may improve recognition and causal assessment of similar cases.

## Data Availability

The original contributions presented in the study are included in the article/**Supplementary Material**. Further inquiries can be directed to the corresponding author.
